# Supplementary material for: Non-Invasive Probing of Winter Dormancy via Time-Frequency Analysis of Induced Chlorophyll Fluorescence in Deciduous Plants as Exemplified by Apple (Malus × domestica Borkh.)
Source: Plants (Basel). 2022 Oct 22;11(21):2811. doi: 10.3390/plants11212811 (PMC9656017; doi:10.3390/plants11212811)
Supplement: Supplementary file 1 [file plants-11-02811-s001.zip › plants-1962834-supplementary.pdf]

plants
MDPI

Supplementary materials for the article “Non-invasive probing of winter dormancy via time-frequency analysis of induced chlorophyll fluorescence in deciduous plants as exemplified by apple (*Malus* × *domestica* Borkh.)” by Boris Shurygin, Ivan Konyukhov, Sergei Khruschev, Alexei Solovchenko

Supplementary methods

1. Sampling scheme

The field site (total area of 0.1 ha) encompassed 400 apple trees, the planting density was 4000 plants/ha. The shoots for indoor incubation experiments were taken from five trees standing in row. These five trees included (i) the two trees from which the Chl fluorescence data were recorded and (ii) one tree between those trees and (iii) the two flanking trees (see Fig. S1).

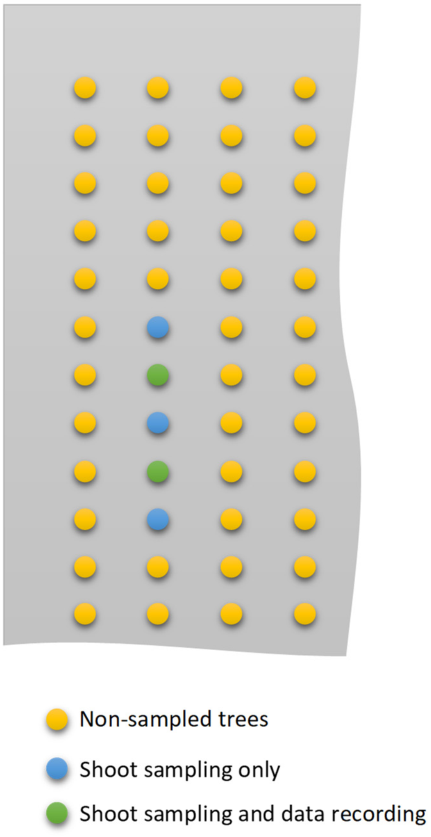

Figure S1. The scheme of experimental tree placement on the field site.

2. The in-house made chlorophyll fluorimeter

Chlorophyll fluorescence signals were monitored by means of a laser fluorimeter built at the department of biophysics, Faculty of Biology, Faculty of Biology, Lomonosov MSU. The device has a transparent watertight measuring chamber made of acryl, and it is implemented with the use of the Fast-Repetition-Rate technology, FRR [61] to induce and to record OJIP fluorescence transients. In our experience, FRR-fluorimetry demonstrates the highest signal-to-noise ratio under the presence of ambient light.

Fluorescence excitation is carried out at a distance of 5 cm from the branch and provided by a red laser diode module with an internal collimating lens (650 nm, 80 mW peak power, Figure S1). We used red excitation light that penetrates deeper into the bark as compared to green or blue light.

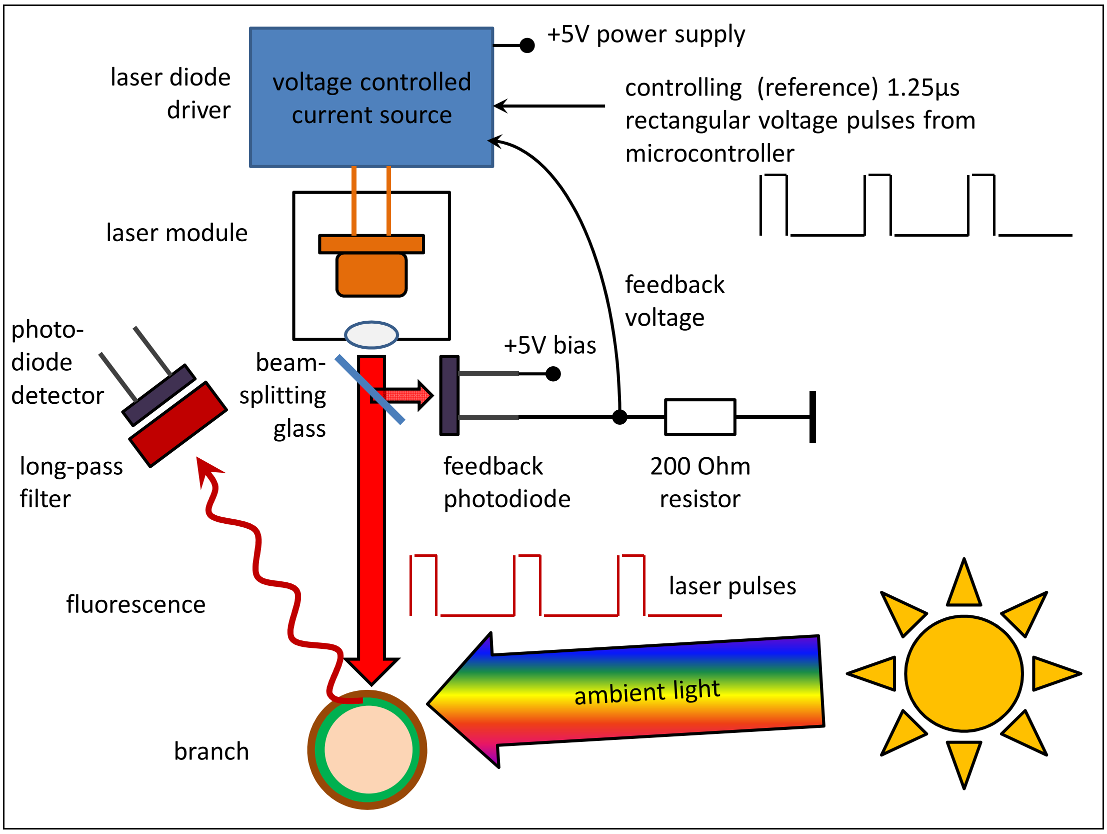

Figure S2. The key components of laser FRR-fluorimeter.

OJIP transient (400 ms long) is induced by a series of identical sub-saturating light pulses (1.25 µs width) following each other at a constant frequency of 200 kHz (Figure S2a). Taking into account a duty cycle of 0.25, the average excitation photon flux density is equal to 20,000 $\mu mol \cdot m^{-2} \cdot s^{-1}$. An auxiliary photodiode and fast optical feedback (Figure S1) are used to stabilize peak laser output power. This feedback compensates lasing threshold current of the diode as well as the influence of reactive components of its electrical impedance which results in good rectangular shape of a single light pulse and sufficient long-term output power stability within an ambient temperature range of –20…+40 °C.

Chlorophyll fluorescence pulses are detected behind a long-pass colored glass filter (two pieces of KS-18 colored glass, Krasnogorsk, Russia) by a silicon p-i-n photodiode with a photosensitive area of 7.5 mm^2. Photocurrent is converted to voltage by a low-noise DC amplifier (f_{-3dB} = 400 kHz). Output voltage level is digitized at a 16-bit resolution. For

synchronous fluorescence detection output signal is sampled twice: at the end of each excitation pulse (1.25 μs after switching on) and prior to the next excitation pulse (≈ 2.5 μs after switching off, Figure S2b). An active fluorescence is quantified as a difference between these two voltage samples (Figure S2b). Synchronous detection completely eliminates the influence of internal voltage offsets of DC amplifier and of ADC, as well as the influence of photodiode dark current and noisy offsets arising from ambient near-infrared light and «passive» chlorophyll fluorescence excited by the sunlight.

The initial point of OJIP curve was used as an approximation of F_t value. F'_m value was found after smoothing the OJIP curve as the maximum fluorescence value within the time interval of 1…400 ms. Effective PS2 photochemical quantum yield under an ambient illumination (Φ'_{PS2}) was calculated as a ratio:

$$\Phi'_{PS2} = \frac{F'_m - F_t}{F'_m} \quad \text{(S1)}$$

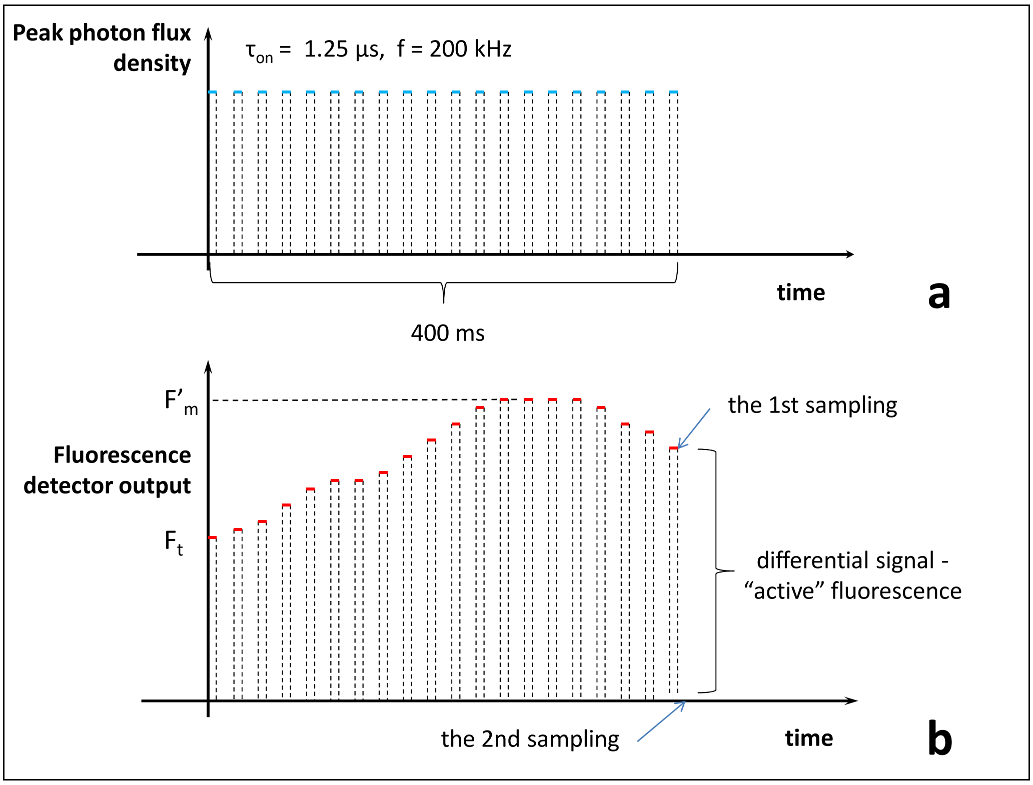

Figure S3. FRR-technology and synchronous fluorescence detection. Schematic timing diagrams of excitation (**a**) and fluorescence (**b**).

Table S1. JIP-test parameters according to (Strasser et al., 2004 [48])

Parameter	Description
F_0	minimal fluorescence, when all PS II RCs are open
$F_K = F_{300\mu s}$	fluorescence level at the K-step (300 μs) of the transient
$F_J = F_{2ms}$	fluorescence level at the J-step (2 ms) of the transient
$F_I = F_{30ms}$	fluorescence level at the I-step (30 ms) of the transient
F_P (= F_m)	maximal recorded fluorescence, at the peak P of the transient
$F_v = F_m – F_0$	maximal variable fluorescence
$V_t = (F_t – F_0)/(F_m – F_0)$	relative variable fluorescence at time t
$V_J = (F_J – F_0)/(F_m – F_0)$	relative variable fluorescence at the J-step
M_0	approximated initial slope (in ms^{-1}) of the fluorescence transient V_t
Area	area between fluorescence curve and F_m
$\varphi_{Po} = F_v/F_m$	maximum quantum yield of primary photochemistry (at t = 0)
$\psi_0 = 1 – V_J$	probability (at t = 0) that a trapped exciton moves an electron into the electron transport chain beyond Q_A

φ_{Eo} = ET_0/ABS = = (1 – F_0/F_m) ψ_0	quantum yield of electron transport (at t = 0)
δ_{Ro} = (1–V_J) (1-V_I)	probability with which an electron from the intersystem electron carriers moves to reduce end electron acceptors at the PSI acceptor side
S_m = (Area)/(F_m–F_0)	normalized total complementary area above the O-J-I-P transient (reflecting multiple-turnover Q_A reduction events)
ABS/RC = = M_0 (1/V_J) (l/φ_{Po})	absorbed light flux per PSII RC
TR_0/RC = M_0 (1/V_J)	trapped energy flux further than Q_A^- per PSII RC (at t = 0)
ET_0/RC = M_0 (1/V_J) ψ_0	electron transport flux per PSII RC (at t = 0)
DI_0/RC = (ABS/RC) – – (TR_0/RC)	dissipated energy flux per PSII RC (at t=0)
RE_0/RC = = M_0 (1/V_J) (1–V_I)	electron flux reducing end electron acceptors at the PSI acceptor side per PSII RC
PI_{ABS} = (RC/ABS) × × (φ_{Po}/(1–φ_{Po})) (ψ_0/(1–ψ_0))	performance index on the absorption basis

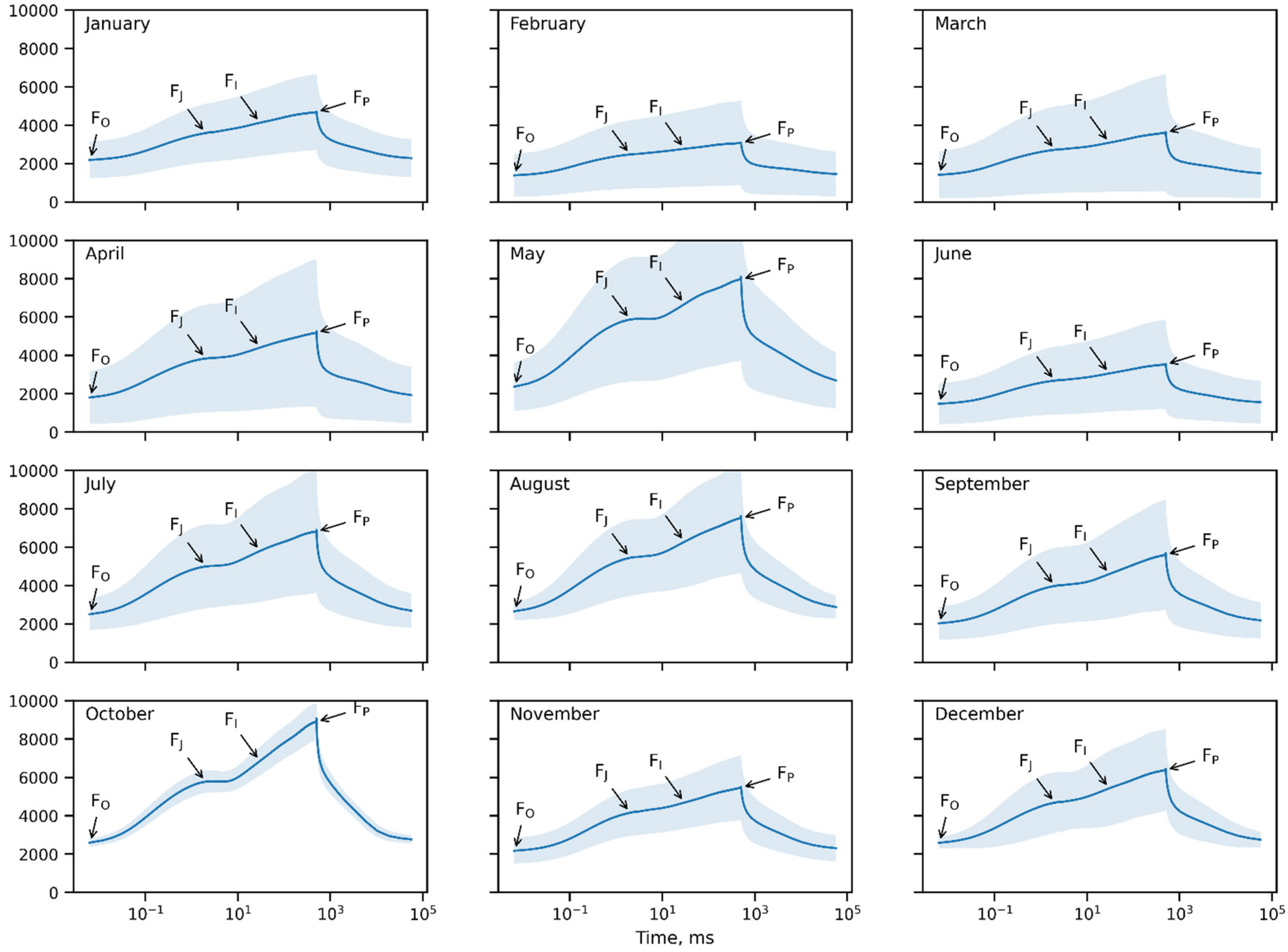

Figure S4. The average CF transients (lines) and their standard deviation (shaded area) measured at night (ten transients recorded around midnight were selected from diel datasets and averaged monthly).

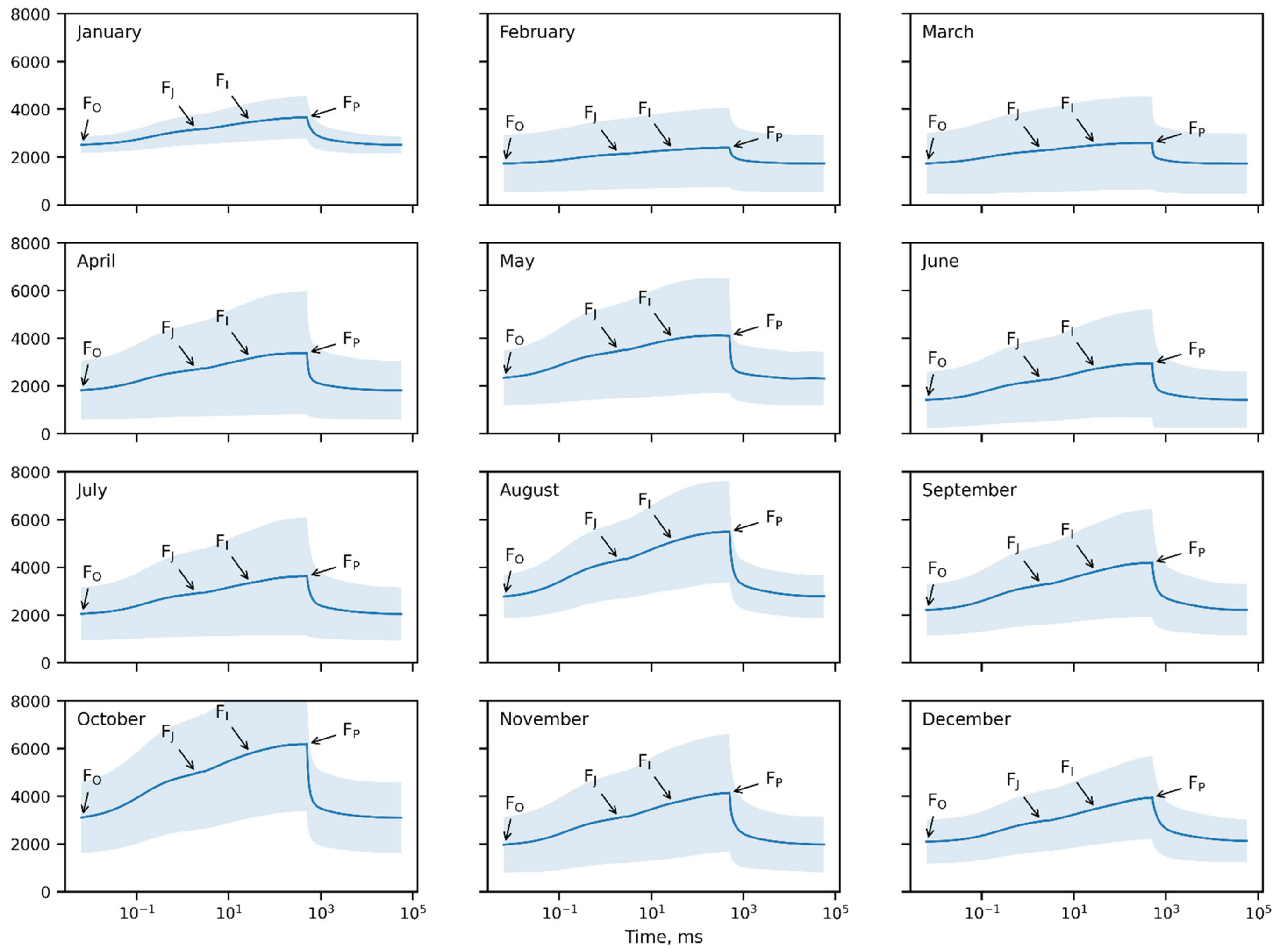

Figure S5. The average CF transients (lines) and their standard deviation (shaded area) measured under daylight conditions (ten transients recorded around midday were selected from diel datasets and averaged monthly).

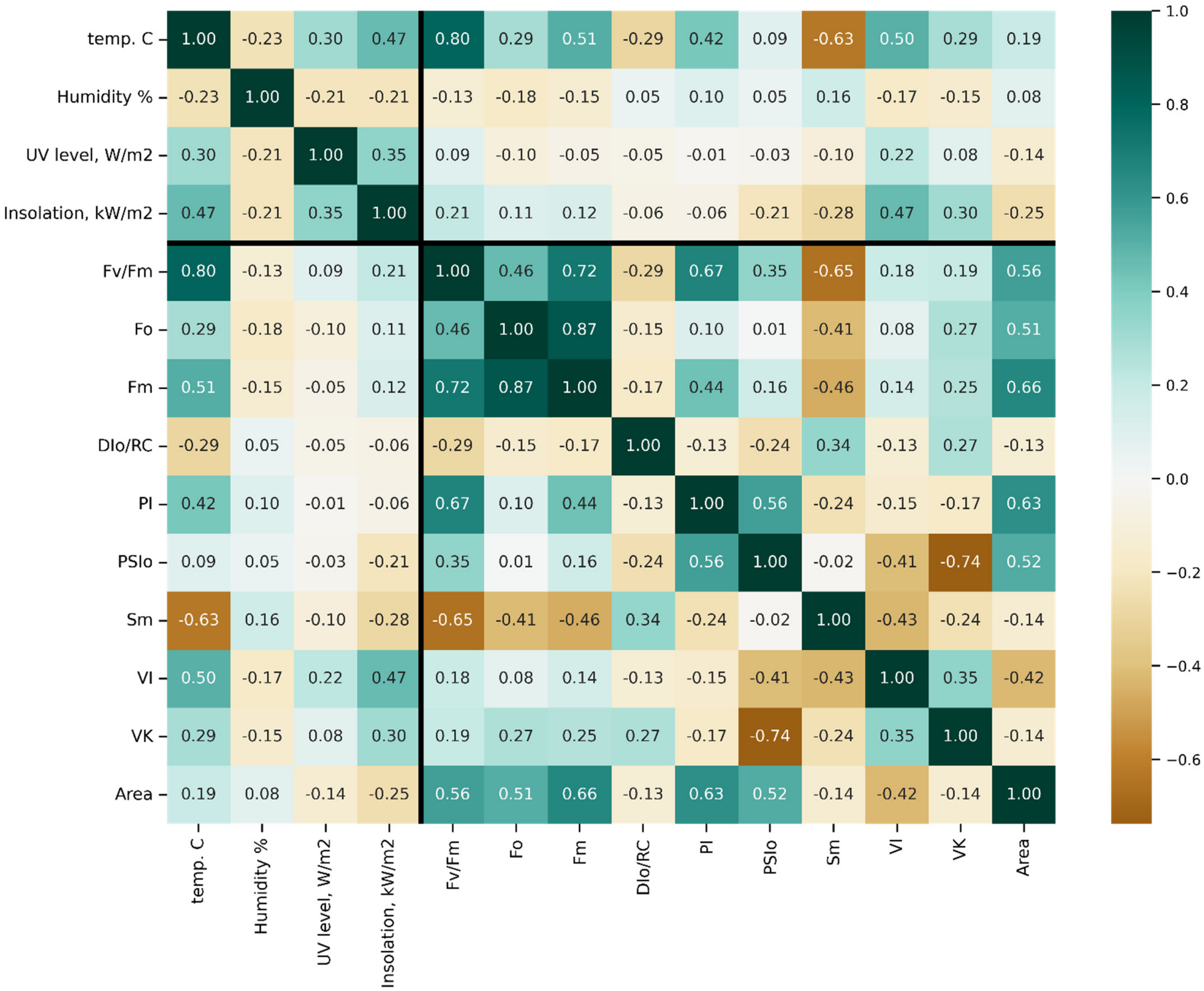

Figure S6. Pearson's *r* value matrix computed for pairs of the parameters studied in this work and calculated on the basis of the CF transients measured under daylight conditions. Thick lines delineate fluorometry from meteorological data. For more detail on JIP test parameters, see also (Strasser et al., 2004 [48]) and Table S1.

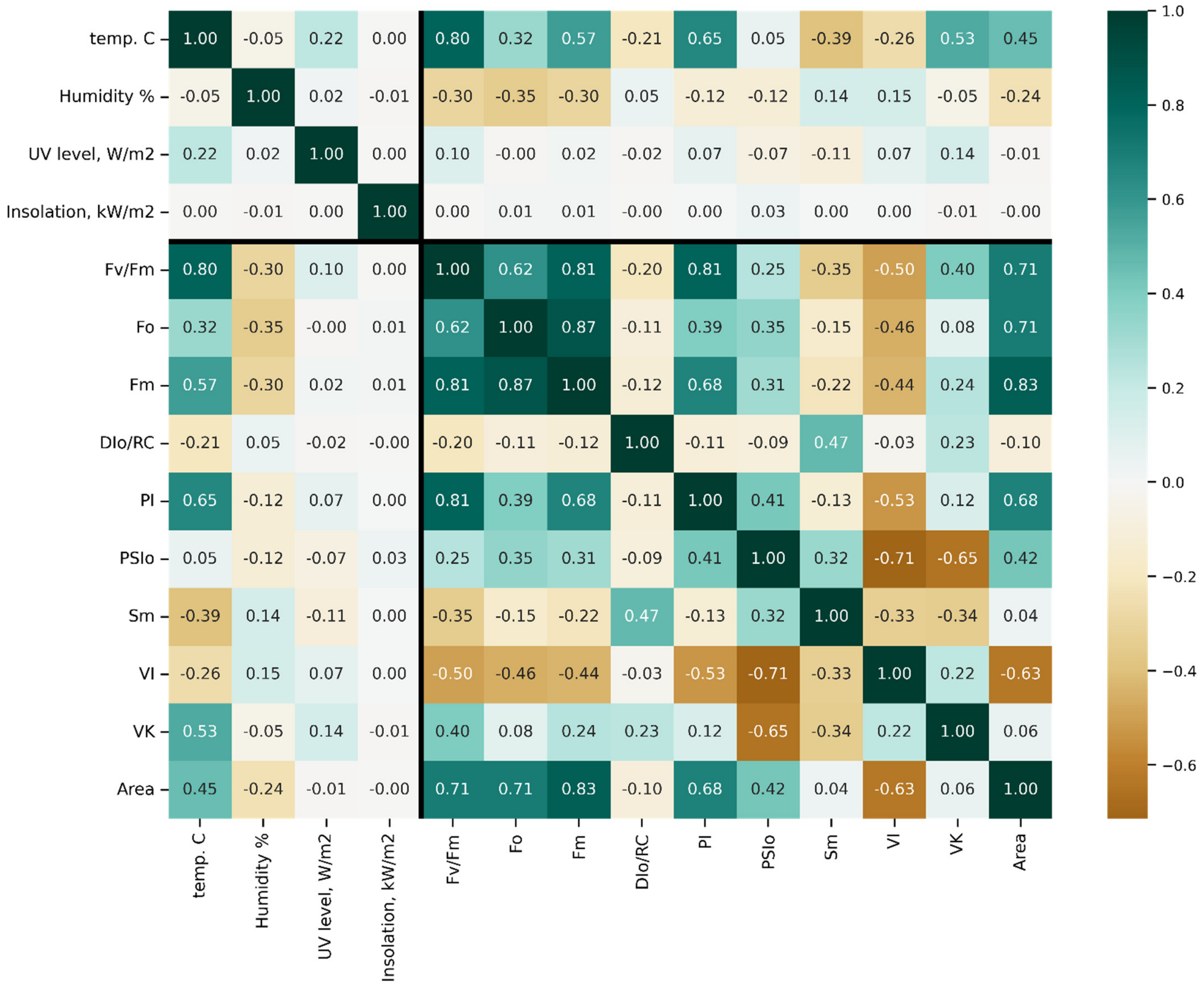

Figure S7. Pearson's *r* value matrix computed for pairs of the parameters studied in this work and calculated on the basis of the CF transients measured under dark conditions (at night). Thick lines delineate fluorometry from meteorological data. For more detail on JIP test parameters, see also [48] and Table S1.

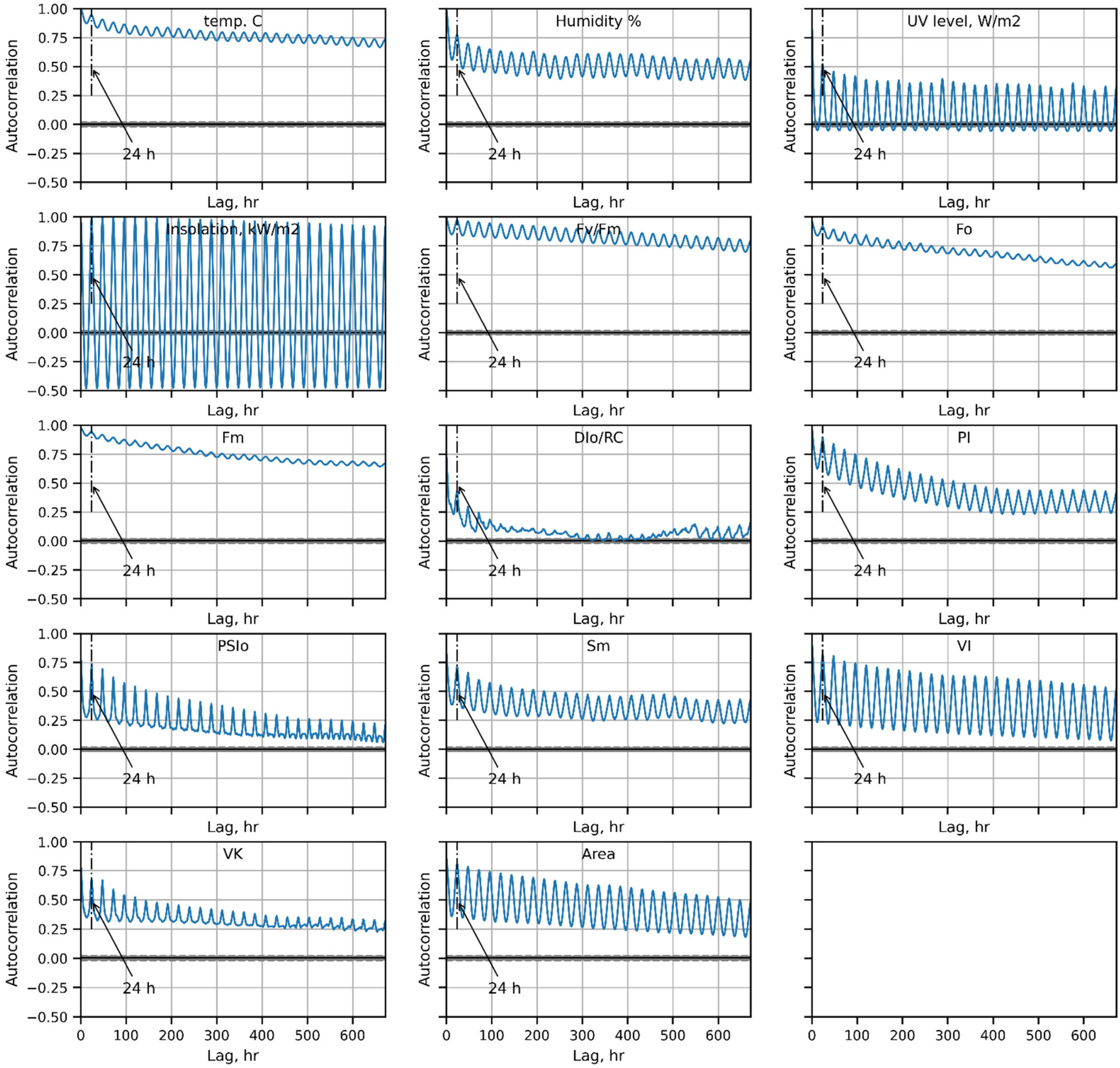

Figure S8. Autocorrelation plots for select JIP-test parameters and insolation estimates. Of note is the comparison between almost perfectly periodic daily cycles of insolation and measured humidity, temperature and Fv/Fm. Autocorrelation was calculated over the entire observation period, thus parameters like temperature are relatively weakly modulated over a timescale of a month. By contrast, PI and DI_0/RC do not exhibit a periodic pattern at all.

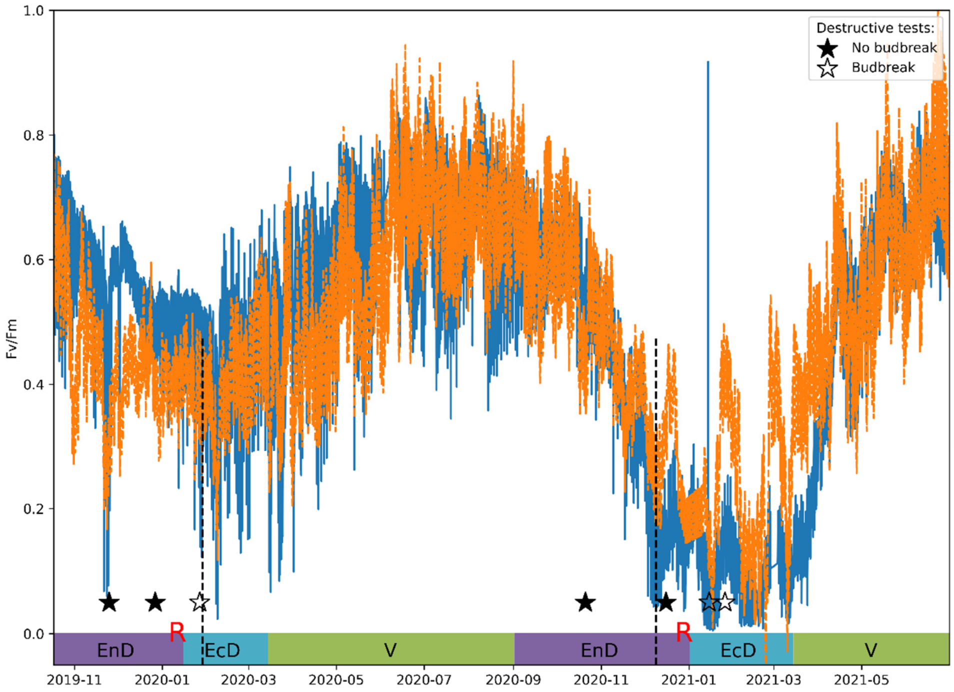

(a)

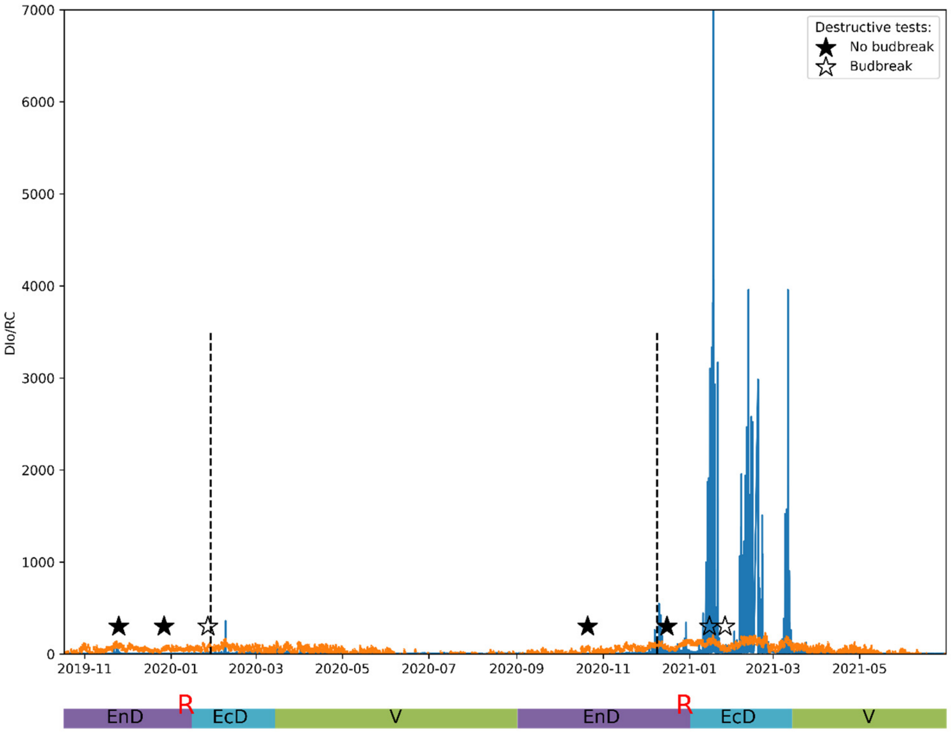

(b)

Figure S9. Multilinear regression fitted curves for (a) Fv/Fm (b) DI_0/RC using only the meteorological data (ambient temperature and insolation).

Theoretical background: time series processing

A vast amount of systems evolving in time could be mathematically described using systems of linear differential equations of first or second order. Solutions to those equations - oscillations, waves, exponential growth and decay - are at the core of nearly every single natural process. Spectral analysis deals with these oscillations in an attempt to reconstruct the system that has produced them. Crucially, the instantaneous values of a periodic process bear little significance, but the overall pattern of vibrations, as described by frequencies and amplitudes, is closely connected to the inner workings of their origin. This information is not readily available in the original signal, however, and has to be extracted first; the most common tool for that, by far, is the Fourier transform.

The Fourier transform converts the function from the time domain to the frequency domain - the temporal information becomes obfuscated and the frequency information revealed. That is, it is readily known when something happens in the original signal, but not what its frequency characteristics are, and for the Fourier-transformed signal the opposite is true. More fundamentally, there is a theoretical limit on simultaneously resolving time and frequency, called the uncertainty principle or the Heisenberg-Gabor limit [69,70]:

$$\sigma_t * \sigma_f \geq \frac{1}{4\pi} \tag{S2}$$

For the time-domain signal, $\sigma_t \to 0$ and $\sigma_f \to \infty$, while for the frequency-domain signal$\sigma_t \to \infty$ and $\sigma_f \to 0$.

To both capture the frequency components and localize them in time, a time-frequency representation (TFR) is required. It trades off time resolution for frequency resolution and vice versa: intuitively, it becomes possible to localize, say, hourly changes within a day and daily changes within a week, a feat next to impossible with the Fourier transform. Notable approaches to this problem include short-time Fourier transform (STFT), Hilbert-Huang transform (HHT), Wigner transform (Wigner distribution function, WDF) and wavelet transform [71]. We are using a wavelet-based approach in this work, which is a time-scale method rather than a time-frequency one, and it could be particularly useful to describe transient processes (idem, p. 21). The Fourier transform could be conceptualized as decomposing the signal into a series of periodic signals, extending indefinitely and unchangeably in time. By contrast, wavelet transform considers a single oscillation or a wave packet, the basis function (also called the mother wavelet), which is localized in time. This function is then scaled and convolved with the original signal - by moving a localized signal in time and comparing it to the initial signal, a measure of the oscillation at the current scale and a given moment in time is taken. Thus, both spectral and temporal components are retained, albeit at a cost of the precision loss. Repeating this procedure allows for describing processes occurring at different time scales.

Mathematically, the Fourier transform of a discrete signal (DFT) is given by

$$F_k = \sum_{n=0}^{N-1} x_n e^{-i2\pi kn/N}, k = 0..N-1, \tag{S3}$$

where x_n is the n-th value of the discrete signal, and F_k is the transformed value corresponding to the frequency k. F_k is complex-valued and normally described in terms of the amplitude and phase, with amplitudes being the prime target for an analysis.

Continuous wavelet transform is given by

$$X_w(a,b) = \frac{1}{|a|^{1/2}} \int_{-\infty}^{\infty} x(t)\psi^*\left(\frac{t-b}{a}\right) dt, \tag{S4}$$

where X_w is the transformed value, a is the scale factor and b is the translation – position in the signal the scaled wavelet is shifted to for piecewise multiplication; ψ is a continuous function called the mother wavelet and a star symbol represents the complex conjugate.

To better illustrate how the two approaches compare to each other, let us consider a toy problem distantly related to the task at hand. Let us assume there are two periodic

processes that can be described as sine waves with different frequencies, and two scenarios involving those processes. In the first instance, both processes are continuous and unchanging, spanning the entire observation. In the second instance, one of the processes only takes place for a fraction of the timespan in which they are being considered. Original signals, as well as their frequency and time-frequency "portraits", are depicted on Supplementary Fig. S9.

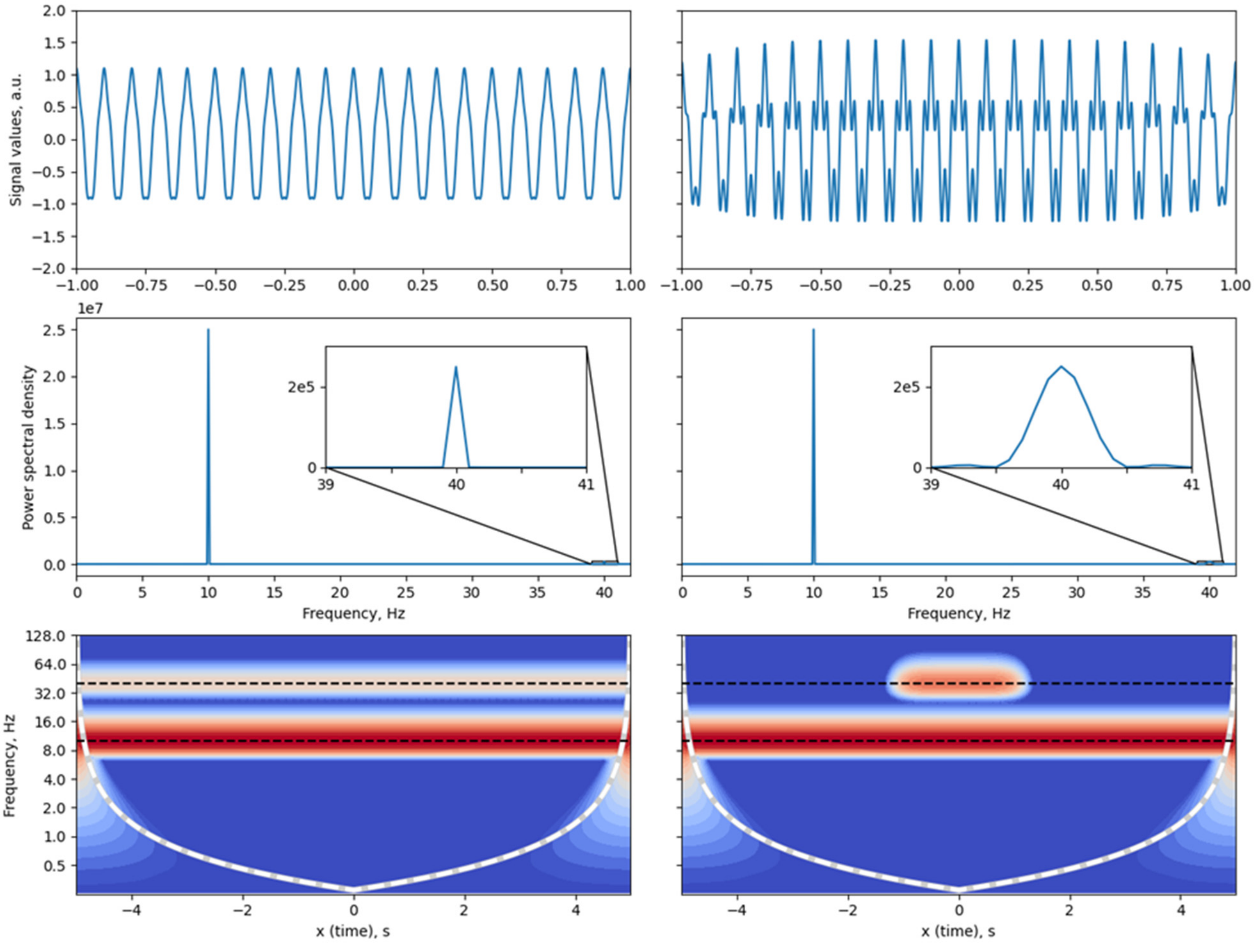

Figure S10. A comparison between Fourier transform and wavelet transform for signal analysis. Top row: a fragment of the original signal. Middle row: Fourier transform of the data. Bottom row: wavelet transform of the same data. Horizontal dashed lines correspond to the frequencies in the model signal (10 Hz and 40 Hz).

The structure of a spectrogram plot begs an additional description. Vertical axis is expressed in either units of frequency or period, and in long-term observations commonly has logarithmic scale. A cone of influence is also plotted (dashed line) - areas outside of it and next to the edges of the plot are affected by processing artifacts and must be excluded from the analysis. These areas exist because the convolution starts operating on incomplete data as the wavelet extends past the boundaries of the original signal - in other words, a description of a weekly trend drawing from only a single day worth of data could not possibly be reliable.

It is clear that for the purposes of qualitative description of the system, time-frequency analysis holds a significant advantage: in fact, there is no easy way to describe the difference between two signals in this example from their Fourier images alone. The main drawback of the wavelet approach could also be clearly seen: a narrow frequency band in

the Fourier spectra appears orders of magnitude wider in the spectrogram, not allowing for as accurate quantitative parameter extraction. In comparison with short-time Fourier transform, wavelet transform provides finer time resolution and coarser frequency resolution for high frequencies and the opposite for low frequencies.

A separation of signal component using the frequency domain is also possible. This allows, among other things, for denoising by filtering out high-frequency component. This kind of filters is called low-pass filters, and typically, they introduces some amount of delay into the filtered signal, which should be compensated for. Among the most commonly used low-pass filters are moving average and moving median filters. Mathematically, they are very efficient at filtering out noise [72], but suffer from the introduced delay being non-deterministic – that is, depending on the frequencies comprising the initial signal, a singular shift value might under- or over-compensate. Since the exact position in time is of concern, we are using finite impulse response (FIR) filters in this work instead: they share many similar characteristics, are slightly less efficient, but fully deterministic (see *idem.*, pp. 229-303).
